# An Efficient and Robust Partial Differential Equation Solver by Flash-Based Computing in Memory

**DOI:** 10.3390/mi14050901

**Published:** 2023-04-22

**Authors:** Yueran Qi, Yang Feng, Jixuan Wu, Zhaohui Sun, Maoying Bai, Chengcheng Wang, Hai Wang, Xuepeng Zhan, Junyu Zhang, Jing Liu, Jiezhi Chen

**Affiliations:** 1 School of Information Science and Engineering, Shandong University, Qingdao 266237, China; 202132729@mail.sdu.edu.cn (Y.Q.); feng.yang@mail.sdu.edu.cn (Y.F.); sunzhaohui@mail.sdu.edu.cn (Z.S.); maoy.bai@mail.sdu.edu.cn (M.B.); 202132732@mail.sdu.edu.cn (C.W.); 201800272037@mail.sdu.edu.cn (H.W.);; 2 Neumem Co., Ltd., Hefei 241060, China; 3 Key Laboratory of Microelectronic Devices and Integrated Technology, Institute of Microelectronics of Chinese Academy of Sciences, Beijing 100029, China

**Keywords:** computing in memory, flash memory, partial differential equations

## Abstract

Flash memory-based computing-in-memory (CIM) architectures have gained popularity due to their remarkable performance in various computation tasks of data processing, including machine learning, neuron networks, and scientific calculations. Especially in the partial differential equation (PDE) solver that has been widely utilized in scientific calculations, high accuracy, processing speed, and low power consumption are the key requirements. This work proposes a novel flash memory-based PDE solver to implement PDE with high accuracy, low power consumption, and fast iterative convergence. Moreover, considering the increasing current noise in nanoscale devices, we investigate the robustness against the noise in the proposed PDE solver. The results show that the noise tolerance limit of the solver can reach more than five times that of the conventional Jacobi CIM solver. Overall, the proposed flash memory-based PDE solver offers a promising solution for scientific calculations that require high accuracy, low power consumption, and good noise immunity, which could help to develop flash-based general computing.

## 1. Introduction

To break the bottleneck of traditional von Neumann architecture, computing-in-memory (CIM) architectures have become a budding technology that can effectively reduce the overhead caused by frequent data transmission between the memory unit and the processing unit [1,2]. So far, most CIM is concentrated in the field of neural networks, such as the edge detection of moving objects by implementing a convolutional neural network (CNN) based on three-dimensional memristors [3], MNIST digit recognition realized on a flash-based deep neural network [4], and an artificial olfactory inference system based on memristive devices [5], while the applications in scientific computing are rarely studied. Scientific computing, such as solving linear equations and partial differential equations (PDE), is a crucial aspect of applied science, social science, and engineering [6,7,8]. Different from neural networks, to implement scientific calculations like the PDE solver, the accuracy and processing speed should be strictly controlled.

In previous work, a high-precision PDE solver in sliced small memristor crossbar arrays was proposed by adopting the Jacobi iterative method and the precision-extension technique [9]; for low-power calculations, mixed-precision architectures, such as the linear equation solver [10] and the PDE solver [11], were proposed by processing high-precision calculations in ALU units and low-precision calculations in CIM units. To enhance the parallel processing speed of large matrix calculations, flash-based CIM as the PDE solver was proposed, and the high accuracy required to solve time-dependent PDE was demonstrated [12]. Additionally, a 32-bit floating-point (FP) CIM architecture was realized on a NOR flash CIM as a solution for general-purpose computation tasks [13]. However, all the above studies used difference pairs or two arrays to handle positive and negative values separately in the calculation. While this method could be effective in achieving the desired outcome, it may also result in greater power consumption.

Different from the mathematical methods to solve PDE, the method that can be utilized in CIM-based PDE solvers should take the hardware factors into account, such as the properties of CIM devices, arrays, and peri-circuits. In these considerations, the Jacobi iterative method was commonly adopted in previous studies because it is simple for the design of CIM operations [14], by which the matrix weights are fixed without the necessity for real-time data updating. However, a large number of iterations by the Jacobi iterative method led to large power consumption and a serious time delay in computing tasks. More importantly, with more iterations, the computing errors caused by the instability of devices and arrays accumulate and seriously affect the final result [15,16,17]. Thereby, for future applications of CIM chips, it is strongly required to optimize the CIM-oriented iteration method and perform the co-optimizations of hardware (the CIM array) and soft strategies.

In this paper, a novel PDE solver for the flash-based CIM array is proposed by adopting the second refinement of the Jacobi (SRJ) iterative method [18], which can reduce the iteration number effectively and optimize the calculation process with improved accuracy. Based on 55 nm flash memory technology, the abilities to solve PDE are demonstrated in different grid situations. In comparison to the standard Jacobi iterative method, faster convergence speed, better anti-noise ability, and lower power consumption are achieved, as well as improved accuracy. Moreover, the pre-processing approach is proposed to save unnecessary array area waste and further suppress the total power consumption.

## 2. Materials and Methods

### 2.1. Architecture

**Figure 1:**
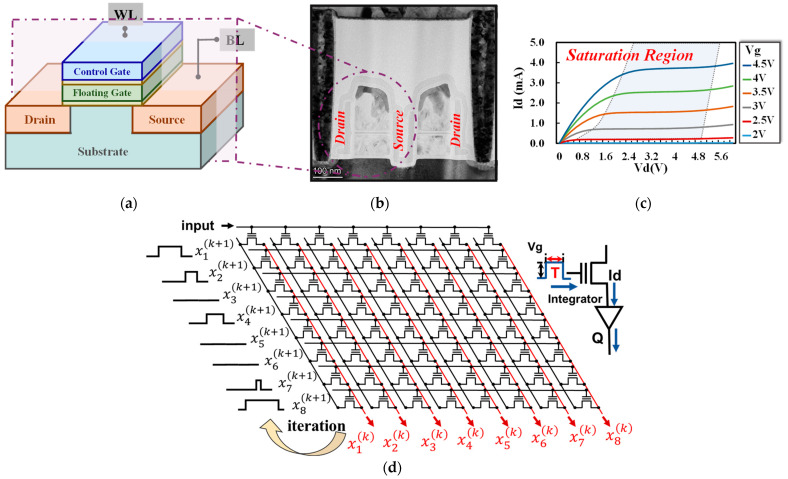
(**a**) Schematic of the structure of a transistor, (**b**) TEM image of the cell’s cross-section, and (**c**) the output characteristic curves are also shown for reference; (**d**) the flash-based PDE solver, input as pulses with variable duration and output as the charge we collected.

**Figure 2:**
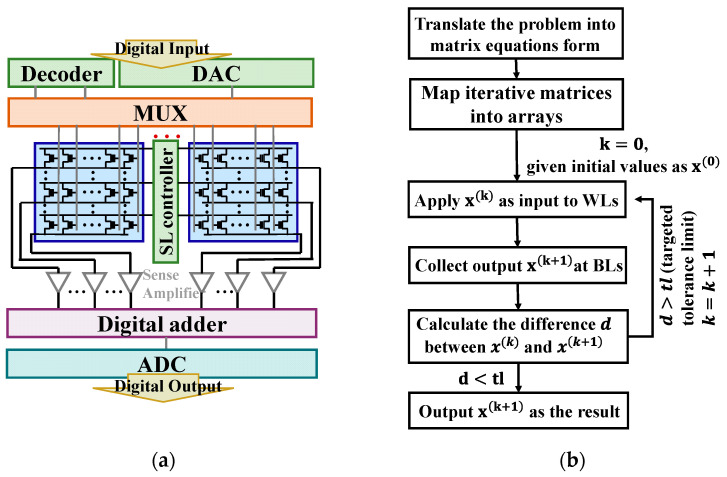
(**a**) Schematic of the system design of the solver; (**b**) the flow chart of the whole solution process.

It is widely acknowledged that data processing has become increasingly demanding in practical applications. The conventional von Neumann architecture results in a significant amount of energy being expended on data transmission between the memory unit and the calculation unit, rather than on computing itself. This can be particularly problematic for certain applications that contain lots of matrix–vector multiplication, such as solving partial differential equations. In such cases, CIM architecture can offer a solution by reducing unnecessary power consumption. Specifically, all the operation processes in this work are littered with large-scale, high-precision multiplication and cumulation (MAC) that we can implement via a one-shot read operation on a flash memory array that has mature technology with capabilities of high bit-density and robust reliability in ultra-large arrays. Figure 1a,b show the stacking structure diagram and the TEM image of one flash memory cell of a 55 nm technology node. Depending on the current characteristics in the saturation region, as shown in Figure 1c, each cell can be considered a variable conductance that can be modulated by the settled gate bias. The entire schematic of the flash memory array as a multiplier is also shown in Figure 1d. In the matrix to process multiplications, the iteration matrix is mapped as the matrix of threshold voltage ($V_{th}$) in the flash memory array, while the vectors are designed by the applied pulses, with pulse durations proportional to the values at a fixed amplitude. In this work, the input (for example, $x_{1}^{\left(k\right)},x_{2}^{\left(k\right)},\ldots ,x_{n}^{\left(k\right)}$) is applied to the word-line (WL), then the multiplication result ($x_{1}^{\left(k+1\right)},x_{2}^{\left(k+1\right)},\ldots ,x_{n}^{\left(k+1\right)}$) is achieved at the source side by integrating the current to obtain the amount of charge according to Ohm’s law and Kirchhoff’s current law. Thereby, we can implement the vector–matrix multiplications with peri-circuits, as presented in Figure 2a.

PDE contains multivariate unknown functions and their partial derivatives of several orders. The Poisson equation is a particular kind of PDE and its main form is shown in Equation (1) [19]. The Poisson Equation (2) exemplified in this work is solved by the finite difference method (FDM), whose main principle is to make a direct difference approximation to the differential term in the equation, translating the differential equation into the linear equation that is easier to be solved.

(1) $$\nabla^{2}u=f$$

(2) $$\{\begin{array}{c} \nabla^{2}u=-2\pi^{2}\sin\pi x\sin\pi y,\;\\ \begin{array}{c} u\left(x,0\right)=u\left(x,2\right)=0,0\leq x\leq 2,\\ u\left(0,y\right)=u\left(2,y\right)=0,0\leq y\leq 2. \end{array} \end{array}$$

To solve PDEs, the first step of the FDM method is to transform the solution domain into discrete grid blocks to obtain the differential approximation of each discrete point. After changing the two-dimensional second-order partial derivatives of x and y into FDM form, it is easy to derive the five-point difference format of the Laplace operator term as

(3) $$\nabla^{2}u=\frac{u_{i+1,j}+u_{i-1,j}+u_{i,j+1}+u_{i,j-1}-4u_{i,j}}{h^{2}}$$

where h is the step size we selected in the x-axis direction, i is the x-axis index of the current grid point, and i−1 and i+1 are the indexes of adjacent grid points. It is the same of j as the y-axis index. Similarly, we choose the same step size of h in the y-axis direction.

**Figure 3:**
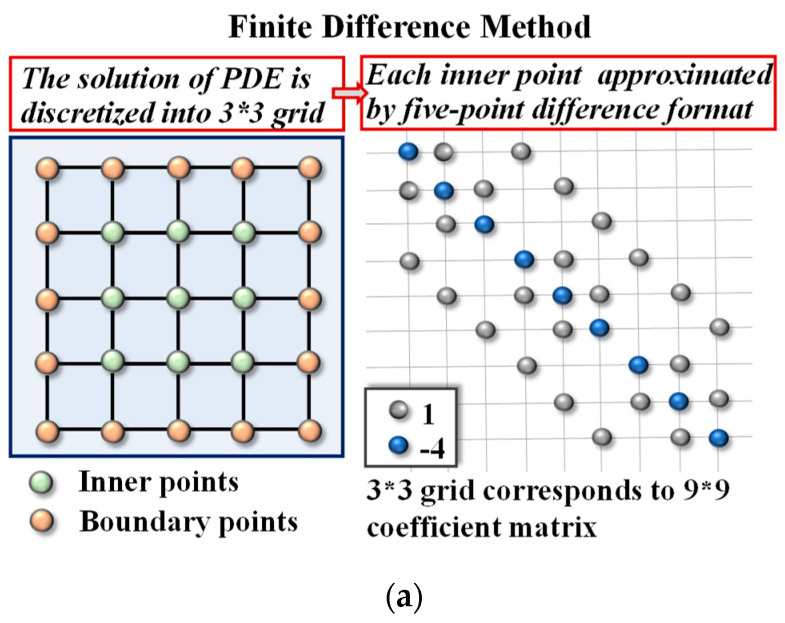

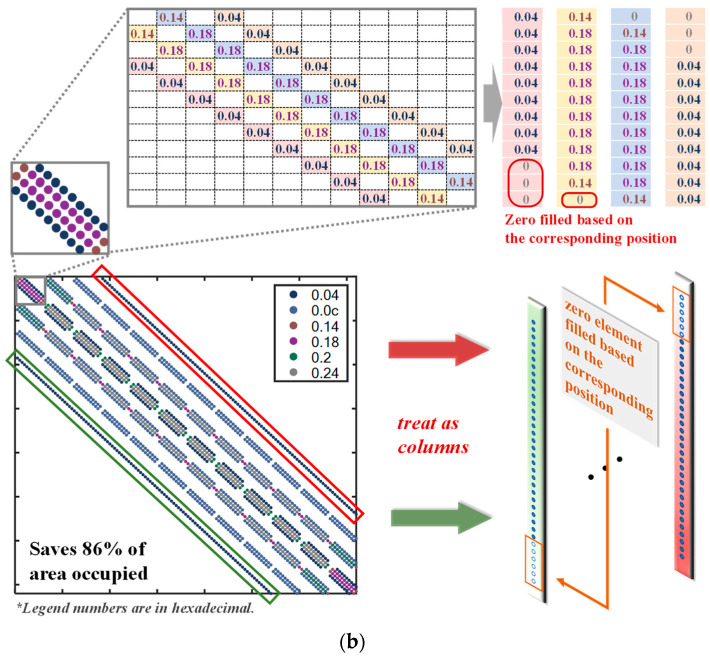
(**a**) The FDM flow (3 × 3 grid) and the point map obtained; (**b**) an example with hexadecimal legend numbers of the SRJ iteration matrix in the case of 12 × 12 grid FDM. Top: the zoomed upper left corner of the matrix is an example to show how the mapping works. Bottom: the first diagonal and the last diagonal chosen as a demonstration are mapped into the array.

To facilitate understanding and determining the function values $u_{i,j}$ of each grid point, we convert the five-point difference format of all inner nodes into a system of difference equations. Then, the left side of Poisson Equation (1), discretized by 3×3 grid points as an example, can be transformed into grid form, as shown in Figure 3a.

Then, we can obtain its matrix equation form as Ax=b,

(4) $$A=\left[\begin{array}{ccccccccc} -4 & 1 & 0 & 1 & 0 & 0 & 0 & 0 & 0\\ 1 & -4 & 1 & 0 & 1 & 0 & 0 & 0 & 0\\ 0 & 1 & -4 & 0 & 0 & 1 & 0 & 0 & 0\\ 1 & 0 & 0 & -4 & 1 & 0 & 1 & 0 & 0\\ 0 & 1 & 0 & 1 & -4 & 1 & 0 & 1 & 0\\ 0 & 0 & 1 & 0 & 1 & -4 & 0 & 0 & 1\\ 0 & 0 & 0 & 1 & 0 & 0 & -4 & 1 & 0\\ 0 & 0 & 0 & 0 & 1 & 0 & 1 & -4 & 1\\ 0 & 0 & 0 & 0 & 0 & 1 & 0 & 1 & -4 \end{array}\right],\;b=\left[\begin{array}{c} h^{2}f_{1,1}+g_{1,0}+g_{0,1}\\ h^{2}f_{2,1}+g_{2,0}\\ h^{2}f_{3,1}+g_{3,0}+g_{4,1}\\ h^{2}f_{1,2}+g_{0,2}\\ h^{2}f_{2,2}\\ h^{2}f_{3,2}+g_{4,2}\\ h^{2}f_{1,3}+g_{0,3}+g_{1,4}\\ h^{2}f_{2,3}+g_{2,4}\\ h^{2}f_{3,3}+g_{3,4}+g_{4,3} \end{array}\right],x=\left[\begin{array}{c} u_{1,1}\\ u_{2,1}\\ u_{3,1}\\ u_{1,2}\\ u_{2,2}\\ u_{3,2}\\ u_{1,3}\\ u_{2,3}\\ u_{3,3} \end{array}\right].$$

where f is the value of the function on the right side of the equation, g is the boundary value given by the boundary conditions. Since we need to obtain the final exact solution by iteration, $x^{\left(k+1\right)}$, obtained from the output value in the previous iteration, is used as the new input in the next iteration. During the process of repeating the output as the next input, the iteration is carried on. When the difference between the result of the previous iteration and the result of the current iteration is below a set value which we call tolerance (tl), the iterative action cuts off. In this case, the output of the last iteration is the final result we obtain. The entire process is described in Figure 2b. In the following studies, we use two different iterative methods for comparisons of their efficiency in obtaining the final exact solution, the standard Jacobi iterative method and the SRJ iterative method.

### 2.2. SRJ Iterative Method

The standard Jacobi iterative method is a generally used method with the format (5) to solve linear equations in the form of A⋅x=b,

(5) $$x^{\left(k+1\right)}=B_{J}x^{\left(k\right)}+f_{J}$$

where $B_{J}=D^{-1}\left(L+U\right)$, $f_{J}=D^{-1}b$, *D* is a diagonal matrix with a non-zero diagonal; *L* and *U* are the upper triangular matrix and lower triangular matrix of *A*, respectively; and A=D−L−U. $x^{\left(k\right)}$ is the $k^{th}$ the iterative result, while $x^{\left(k+1\right)}$ is the ${\left(k+1\right)}^{th}$.

In this work, considering the convergence speed, we use a new modified algorithm of Jacobi instead: the second-refinement of Jacobi (SRJ) iterative method. The iteration format of SRJ is

(6) $$x^{\left(k+1\right)}={\left[D^{-1}\left(L+U\right)\right]}^{3}x^{\left(k\right)}+\left[I+D^{-1}\left(L+U\right)+{\left(D^{-1}\left(L+U\right)\right)}^{2}\right]D^{-1}b$$

and the simplified form can be expressed as

(7) $$x^{\left(k+1\right)}=B_{J}{}^{3}x^{\left(k\right)}+\left(I+B_{J}+B_{J}{}^{2}\right)f_{J}$$

where I is an n-dimensional identity matrix.

To accelerate the iterative process, we can use the result $x^{\left(k+1\right)}$ twice in Formula (5) to obtain the final optimized iterative Formula (7), then the results of three Jacobi iterations can be achieved in a single SRJ iteration process. It is critical for CIM-based PDE solvers that the increase in the number of iterations inevitably leads to an increase in power consumption. In order to reduce the power consumption, the tradeoff between accuracy and the number of iterations needs to be taken into consideration to ensure the optimal CIM PDE solver solution with low power consumption and latency.

### 2.3. Pre-Processing

Although the values of the iteration matrix are all positive, there is a tendency that intermediate results during the iteration, that is, the input of the next iteration, could have negative values, which is contrary to the requirement that input values cannot be negative due to the physical significance of flash memory. To fix this problem, we normalize the input value to a positive value with the form of (8) instead of using two different flash arrays to handle negative and positive values, respectively. This can reduce the array area to half. The pre-processing part is implemented in the software and applied to the arrays by DAC operation to make sure that all values in the array are positive to match the physical requirements of the flash memory.

(8) $$x_{new}=\frac{x_{original}-x_{min}}{x_{max}-x_{min}}$$

Then, the original output result can be easily obtained by calculating the output result gained by pre-processing via the inverse normalization formula (9),

(9) $$y_{original}=\left(x_{max}-x_{min}\right)\cdot y_{new}-D\cdot x_{min}$$

where $y_{new}$ is the output after pre-processing, $y_{original}$ is the correct result that should have been obtained, and D is the matrix value stored in the array. Additionally, the inverse normalized output, that is, the actual solution $y_{original}$ during this iteration, can be reconstructed after ADC transforms the output of integrators into digital form. Then, if the iteration does not converge, that is the solution does not reach a stable state, the solution would be reapplied as the input of the next iteration to achieve the final result. 

### 2.4. Mapping Method

Since the SRJ iterative matrices and Jacobi iterative method are both characterized by their large and sparse identity, the zero elements predominate in the matrix. Therefore, we can use a partition method as indicated in Figure 3b to map the matrix to a flash memory array rather than mapping all elements with large amounts of zero elements that will cause excessive area waste. To pull all the non-zero diagonals into columns and fill in the zeros above or below, according to the corresponding positional relationship, this method maximizes array utilization while reducing array area and prevents most invalid zero weights in the flash array. Therefore, in large-scale computing with high-sparsity data, adapting this mapping method can significantly improve system performance and ensure that the memory array operates efficiently and effectively.

## 3. Results

**Figure 4:**
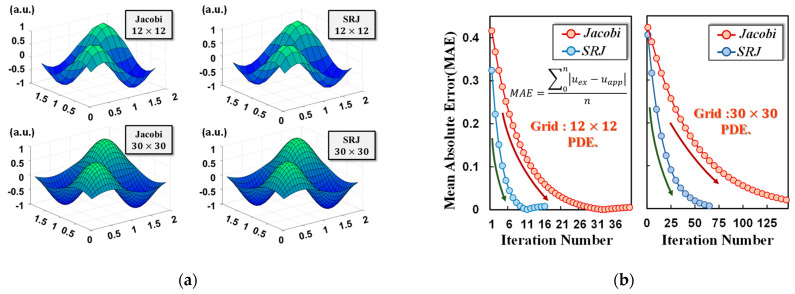

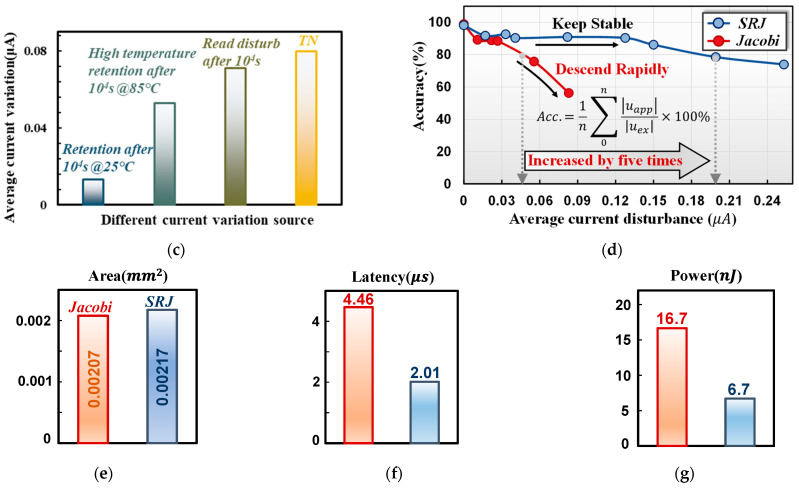
(**a**) Image of the approximate solution of Poisson Equation (2) from the flash memory solver using the Jacobi iterative method (**left top**) and the SRJ iterative method (**right top**) with a 12 × 12 grid; and the Jacobi iterative method (**left bottom**) and the SRJ iterative method (**right bottom**) with a 30 × 30 grid. (**b**) Trend graphs of the MAE values in solving (**a**) PDEs with 12×12 grid and (**b**) PDEs with 30×30 grid. The orange/blue stands for Jacobi/SRJ, respectively. $u_{ex}$ in the formula is the exact result by MATLAB, and $u_{app}\;$ is the approximate iterative solution. The iteration termination condition for both methods is the tolerance of $1\times 10^{-3}$. (**c**) The current summary of current variations in different tests. (**d**) The variation of accuracy with the average current disturbance increasing. (**e**) Estimated area, (**f**) latency, and (**g**) power by Jacobi CIM and SRJ CIM when solving Poisson’s equation with a 12×12 grid.

Based on the aforementioned optimization methods, we studied the performances of the Jacobi method, which has been widely applied in previous studies, and the new SRJ iterative methods in solving PDEs in 32-bit fixed-point precision. Here, the flash model we used to simulate is the commercial 55 nm NOR flash, which has a native 4-bit precision. To overcome this limitation, the final solution is computed by digital adders according to the precision-extension technique [9]. To verify the influence of the grid number on the accuracy, 12×12 is used as the number of grid points to solve the equation by two different iterative methods. The tolerance (tl) that is described as the iteration break condition in Figure 2b is $1\times 10^{-3}$ in the simulation. The results obtained by Jacobi and SRJ are plotted in the top part of Figure 4a. As a comparison, we selected denser grid points (30×30) to obtain more accurate results, and the results are plotted in the bottom part of Figure 4a. It is noticed that the iteration number by SRJ is just one-third of that by Jacobi, which can bring a reduction in power consumption.

In addition to the aforementioned statement, we proceeded to perform a comparative analysis of the two methods, focusing on three key aspects: precision, robustness performance to noise, and power dissipation. The results are presented in the following section.

### 3.1. Accuracy Analysis

The mean absolute error (MAE) values of each iteration are recorded during the solving process and made into trend graphs, as shown in Figure 4b. The exact solution of this equation is given by the MATLAB internal solver. It is revealed by the trend graph that the MAE value of the SRJ decreases extremely fast at the beginning of the iteration, regardless of whether the case is a 12×12 grid or a 30×30 grid. These phenomena indicate that the SRJ solver converges rapidly in all cases, demonstrating the efficiency of the SRJ method in achieving accurate results with fewer iterations. This means that much more power consumption can be effective by adopting the SRJ method for scientific computing tasks. It is noticed that the precision of the Jacobi solver decreases dramatically when the grid mesh becomes dense, even more so than the results of the SRJ solver in a rough grid situation, contrary to the assumption that a finer mesh should be closer to the exact solution. Nevertheless, the SRJ solver fits well with the idea regardless of the case, showing that the MAE value approaches zero as the mesh becomes finer. Thus, we can conclude that the SRJ solver can perform better with denser grid conditions; in other words, it will be better when the target solution is closer to the true value.

### 3.2. Robustness Performance to Noise

The current variation is the key factor determining computing accuracy, which is affected by external conditions and devices. To evaluate the impact of current variations, aspects of reality that affect flash memory reliability, including retention, read disturb, and telegraph noise (TN), are summarized in Figure 4c. The average current drifts after $1\times 10^{4}$ s of retention both at 25 °C and 85 °C are smaller than read disturbance and TN. However, the current variations remain under control, indicating the capability of flash memory to construct the large array system.

To explore the stability of the architecture, the effects of the current disturbance on the accuracy are tested. Figure 4d shows the comparison of the Jacobi solver and the SRJ solver in a 12×12 grid condition. When the average current disturbance reaches 0.04 μA, the accuracy of the Jacobi solver dropped down to 80%, which is fatal for functions that need a high accuracy, such as solving PDEs, while at the same time, the SRJ solver still performs well even at 0.2 μA, which is five times the tolerance limit of the Jacobi solver, and the accuracy is maintained above 80%. It is noticed that current variations remain in a controlled range without accuracy loss, according to Figure 4d. Thus, the SRJ solver can achieve a better robustness compared to the traditional Jacobi solver.

### 3.3. Power Dissipation

To analyze the power dissipation of the CIM architecture, the iteration number and precision of this PDE solver architecture are evaluated by making a comparison between two iterative methods. The simulation results show that only 16 iterations are needed by the SRJ method when processing PDE with a 12×12 grid, which is much lower than the 40 iterations by the Jacobi method. Similar results are also obtained in the PDE with a 30×30 grid. The iteration number of the SRJ method and the Jacobi method are 67 and 147, respectively. The MAE of the SRJ architecture with a 30×30 grid is 0.005, much smaller than the MAE of the Jacobi architecture (0.019). More nonzero values in the SRJ method make the array power dissipation of the SRJ architecture larger than the Jacobi architecture, which is 30 pJ and 15 pJ, respectively, in one PDE solution with a 12×12 grid. 

Moreover, the power dissipation containing peripheral circuits must also be considered [20]. The traditional peripheral circuit of the PDE solver can be directly used in the proposed PDE solver without any modification, and the specific peripheral circuit parameters refer to [21]. The switch matrix controls multiple word lines (WLs) and bit lines (BLs), and the read circuit is used to convert analog current to digital output. An adder and subtractor are also needed to realize the entire computing process. Similar to other CIM applications, write pulses much shorter than traditional write operation are required to tune the memory states with higher accuracy. The results of peripheral circuits in Figure 4e–g are simulated results based on the NeuroSim simulation tool [21], which can be utilized for the estimations of recognition accuracy and power consumption [22,23]. As shown in Figure 4e–g, although the area of the array and peripheral circuit in the SRJ architecture is slightly larger than the Jacobi architecture because the iteration matrix of the SRJ method is somewhat larger, the simulation results shown in Figure 4e confirm that the latency of the Jacobi method is over two times higher than that of the SRJ method when solving a Poisson equation. This provides evidence that the SRJ mapping scheme exhibits exceptional performance, with an operating speed that is more than twice as fast as that of the Jacobi method. Simultaneously, the power consumption can also be well suppressed by the reduction of iteration numbers.

## 4. Discussion

So far, several PDE solvers has been proposed with the CIM architecture for efficient computing. Compared with the previously proposed method to solve the matrix equation in one step by sacrificing the accuracy [24], our architecture accelerates computation processes with the premise of maintaining calculation accuracy. Especially for the high-precision required by PDE solvers, accuracy and reliability are the most important concerns. While taking the feasibility for large-matrix processing into account, the noise immunity, array size, power dissipation, as well as the convergence speed are all important and co-optimizations are strongly required. Therefore, the proposed flash-based PDE solver could provide a feasible approach because flash memory is a mature technology with capabilities of high bit density in ultra-large arrays and robust reliability that can carry out large-scale high-precision computing tasks. By adopting flash technology, we need to design the processing approach for the triple-terminal cell and optimize the operation schemes to the unique properties and array designs of flash memory, as described in Figure 1 and Figure 2. Moreover, in this work, we propose to solve PDEs by using the SRJ method, which makes a distinction between our work and other related work. Table 1 shows the comparison between our work and other prior CIM solvers. Specifically, the algorithm in [9,13,25] is the Jacobi method, in [8,11] it is the residual method, and in [26] it is the fourth order Runge–Kutta method. Different algorithms lead to different results. As can be seen, the proposed PDE solver can maintain a high accuracy and low residual norm errors together with a low power dissipation.

## 5. Conclusions

To sum up, we propose a flash-based CIM architecture as the PDE solver by adopting the SRJ iterative method. Due to the fast operation speed and large storage capacity of flash memory, high-speed data processing and high-precision computing results can be achieved in a large-scale flash memory array. Moreover, an efficient iterative algorithm, SRJ, is proposed to minimize iteration times and accelerate the solving process. Compared with the standard PDE solver using the Jacobi iterative method, this architecture can largely suppress power consumption with much improved calculation accuracy and current noise tolerance. The influence of the array size (grid points) on the computing accuracy is also investigated, showing the ability of the SRJ architecture to achieve high-accuracy solutions for fine grids. Furthermore, the analysis of peripheral circuits in the system indicates that co-optimizations of CIM hardware (the memory cells and the array) with the soft architectures are the key to developing general-purpose CIM applications. This work provides a feasible flash-based CIM that has great potential to overcome the limitations of traditional von Neumann architectures and enable a high-precision and high-performance PDE solver. This could help pave the way for the further development of CIM in general computing.

## Figures and Tables

**Table 1:** Comparison with the state of the art.

	CIM Device	Power Dissipation	Accuracy	Residual Norm Error	Latency
This Work	NOR Flash (55 nm)	6.7 nJ	98.78%	$2\times 10^{-8}$	2.01 μs
Gallo [10]	PCM (90 nm)	<1−100 fJ per device	---	$\sim 1\times 10^{-6}$	<100 ns
Zidan [9]	RRAM	---	>97.3%	---	1 μs
Chen [25]	SRAM (180 nm)	---	---	---	90 ns
Feng [13]	NOR Flash (65 nm)	---	100%	---	---
Ensan [26]	RRAM (65 nm)	---	97%	---	25 ns
Yang [11]	RRAM (22-nm) &CPU/GPU	30 mJ	---	$8\times 10^{-15}$	---

## Data Availability

The data presented in this study are available on request from the corresponding author.
